# Supplementary material for: Microwave Modification of Quinoa Grains at Constant and Varying Water Content Modulates Changes in Structural and Physico-Chemical Properties of the Resulting Flours
Source: Foods. 2023 Mar 27;12(7):1421. doi: 10.3390/foods12071421 (PMC10093526; doi:10.3390/foods12071421)
Supplement: Supplementary file 1 [file foods-12-01421-s001.zip › foods-2249792-supplementary.pdf]

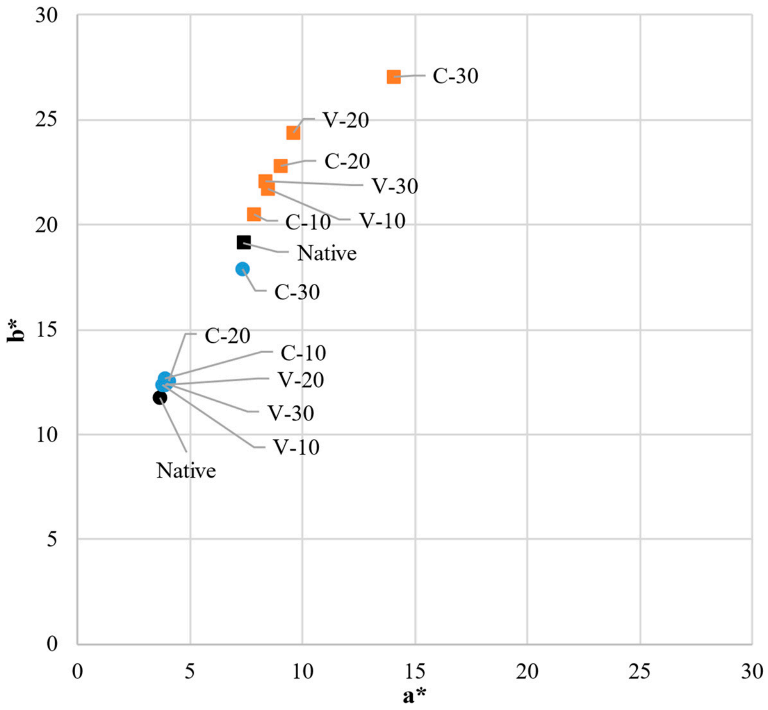

Figure S1. a*-b* chromatic diagram of native (black) and microwaved-quinoa grains (orange, ■) and of flours obtained after milling the grains (blue, ●). V-10, V-20, V-30, C-10, C-20, and C-30: samples obtained from microwaved quinoa grains with 10%, 20%, and 30% water content, in systems with variable (V) and constant (C) water content, respectively.

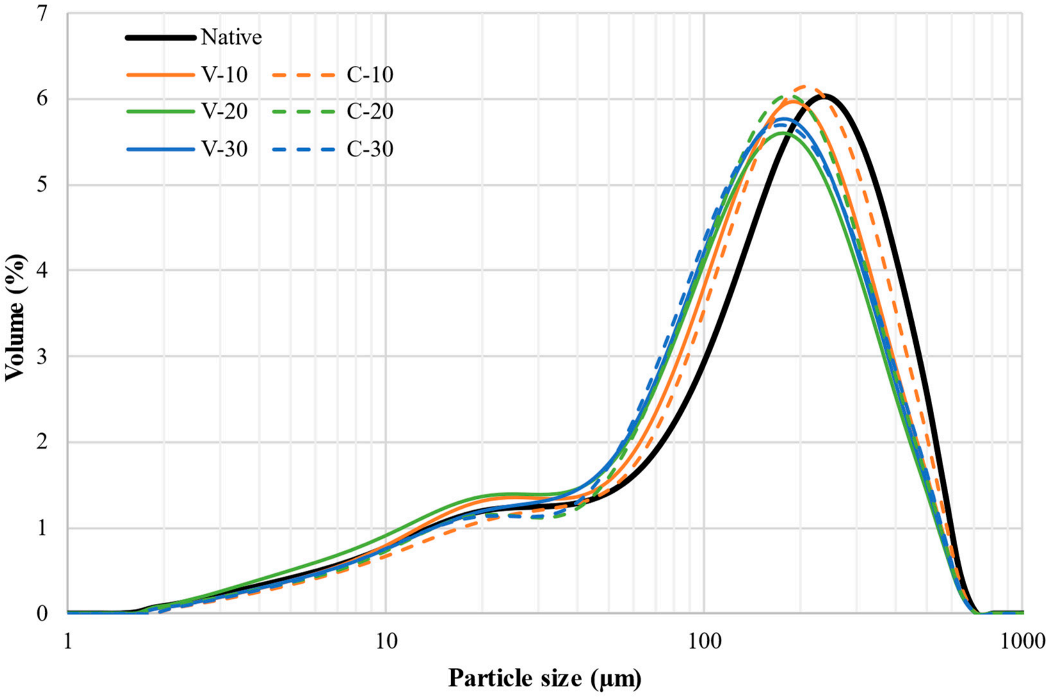

Figure S2. Particle size distribution of untreated flour (Native) and flours obtained from microwaved grains with 10, 20, and 30% water content in systems with variable (V) and constant (C) water content.

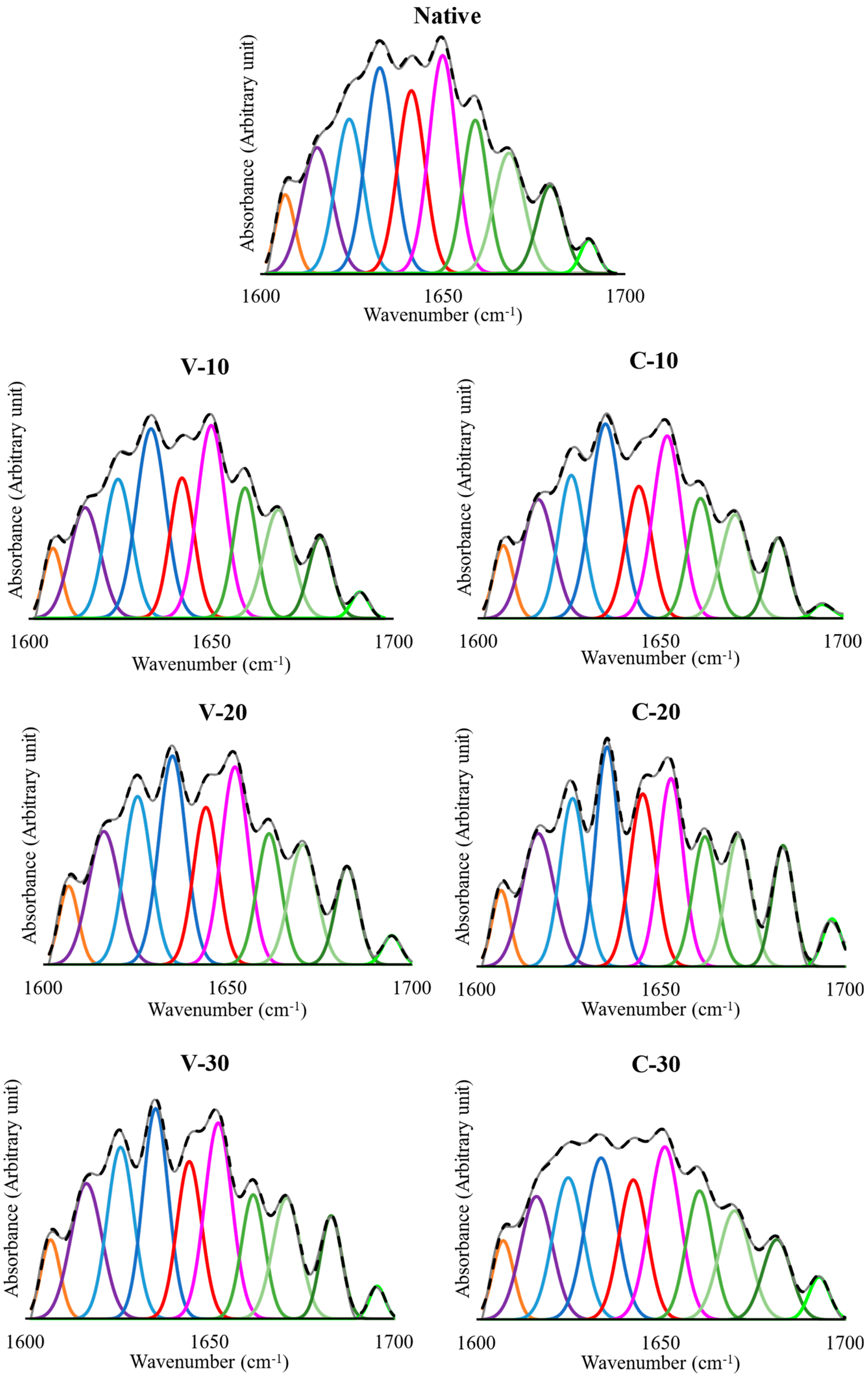

Figure S3. Deconvolved Amide I bands of untreated flour (Native) and flours obtained from microwaved grains with 10, 20, and 30% water content in systems with variable (V) or constant (C) water content. Curve fitting carried out assuming Gaussian band shapes for deconvolved components. The deconvolved FTIR spectra is represented by a grey solid line, and the cumulative fitted curve by a black discontinuous one.
